# Process and implementation of Audio Computer Assisted Self-Interviewing (ACASI) assessments in low resource settings: a case example from Zambia

**DOI:** 10.1017/gmh.2016.19

**Published:** 2016-08-12

**Authors:** J. C. Kane, L. K. Murray, S. Sughrue, J. DeMulder, S. Skavenski van Wyk, J. Queenan, A. Tang, P. Bolton

**Affiliations:** 1 Department of Mental Health, Johns Hopkins Bloomberg School of Public Health, 624 North Broadway, Baltimore, MD, 21205, USA; 2 Department of Public Health and Community Medicine, Tufts University School of Medicine, 136 Harrison Avenue, Boston, MA 02111, USA; 3 Department of International Health, Johns Hopkins Bloomberg School of Public Health, 615 North Wolfe Street, Baltimore, MD, 21205, USA

**Keywords:** ACASI, low- and middle-income country, adolescent, assessment methods, HIV

## Abstract

**Background.:**

Studies from low- and middle-income countries (LMIC) indicate that the use of audio computer-assisted self-interviewing (ACASI) is associated with more accurate reporting of sensitive behaviors (e.g. substance use and sexual risk behaviors) compared with interviewer-administered questionnaires. There is a lack of published information on the process of designing, developing, and implementing ACASI in LMIC. In this paper we describe our experience implementing an ACASI system for use with a population of orphans and vulnerable children in Zambia.

**Methods.:**

A questionnaire of mental health, substance use, and HIV risk behaviors was converted into an ACASI system, tested in pilot and validity studies, and implemented for use in a randomized controlled trial. Successes, barriers, and challenges associated with each stage in the development and implementation of ACASI are described.

**Results.:**

We were able to convert a lengthy and complex survey into an ACASI system that was feasible for use in Zambia. Lessons learned include the importance of: (1) piloting the written and electronic versions; (2) proper and extensive training for study assessors to use ACASI and for those doing voice recordings; and (3) attention to logistics such as appropriate space, internet, and power.

**Conclusions.:**

We found that ACASI was feasible and acceptable in Zambia with proper planning, training, and supervision. Given mounting evidence indicating that ACASI provides more accurate self-report data and immediate data download compared with interview-administered measures, it may be an effective and economical alternative for behavioral health research studies in LMIC.

## Background

The HIV epidemic in sub-Saharan Africa has caused over 15 million children to be orphaned or made otherwise vulnerable, a number that is expected to increase in the near future (UNICEF, 2013). Orphans and vulnerable children (OVC) are at an increased risk for substance use, HIV risk behaviors, functional impairment, and poor psychosocial outcomes (Boris *et al.* 2008; Cluver & Orkin, 2009; Pufall *et al.* 2014). Social desirability and interviewer biases are significant concerns and potential impediments to the collection of valid data on these sensitive outcomes (Ghanem *et al.* 2005).

Audio computer assisted self-interviewing (ACASI) was developed originally for use in high income countries to help reduce these biases and increase accuracy of reporting from study participants (Aquilino, 1997; Turner *et al.* 1998; Des Jarlais *et al.* 1999; Metzger *et al.* 2000). ACASI-administered surveys allow participants to complete interviews on their own without the presence of a human interviewer. Questions and response options are displayed as text on computer screens and simultaneously read aloud to the participant through headphones (http://acasi.tufts.edu/; Tufts University School of Medicine, 2014). The benefits of ACASI-based interviews relative to other modalities, such as self-administered questionnaires and face-to-face interviewing include: (1) increased data validity for sensitive measures due to elimination of social desirability and interviewer biases; (2) increased participant privacy; (3) functionality for illiterate participants; (4) ability to be programmed in multiple languages; (5) automatic and accurate programming of skip/logic patterns; (6) fewer missing data and better non-response rates; (7) no effect of inter-rater variance (8) reduction in staff time needed for interviewing; and (9) obviation of the need for costly, time-intensive and (possibly) inaccurate data entry (Van de Wijgert *et al.* 2000; Brown *et al.* 2013).

ACASI may also be a more economical method of data collection if the system is built for use in ongoing clinical research or can be used in multiple research studies. A cost effectiveness investigation conducted in the USA by Brown *et al*. (2008) suggested that, although start-up costs for ACASI design, software, and hardware may make it more initially expensive than self-reported questionnaire methods, if ACASI is used for multiple studies or programs its costs are spread over several projects and a longer period of time (Brown *et al.* 2013, 2008). Furthermore, because the primary costs of ACASI are upfront expenditures associated with building the system, it is particularly cost effective for studies or programs that assess large numbers of participants or clients because there are no additional data entry charges (in time or personnel) (Brown *et al.* 2013).

A review by Langhaug *et al*. (2010) identified 26 studies of sexual behaviors conducted in low- and middle-income countries (LMIC) examining the use of ACASI as compared with other interview techniques. The review found strong evidence that ACASI-administered interviews resulted in lower rates of reporting bias than comparison methods, including self-administered questionnaires and face-to-face interviewing. More recent studies published after the review have found similar results (Beauclair *et al.* 2013; Adebajo *et al.* 2014). Although the majority of studies on ACASI in LMIC have focused on measures that include items on sexual behavior, research also suggests that reporting of mental health symptoms may be more accurate using ACASI. Langhaug *et al.* (2009) found that the prevalence of common mental disorders was higher and the occurrence of missing data lower when interviews were conducted with ACASI compared with face to face.

The findings from these studies in LMIC have demonstrated the promise of ACASI in improving data quality, however, most of the reports only provide general information on the actual set-up and feasibility of ACASI itself (Langhaug *et al.* 2010). The specific feasibility challenges associated with ACASI in LMIC are often not delineated.

In this paper we describe the process of developing, testing, and implementing an ACASI system for use with an OVC population in Zambia in 2013. Zambia is classified as a lower middle-income country by the World Bank, with a life expectancy of 60 years, and a gross national income per capita of $1680 (World Bank, 2015). The setting for the ACASI development and piloting described in this paper was in Lusaka, the capital city of Zambia. Specifically, study activities took place in ‘compounds’ in Lusaka, low socioeconomic areas with high density populations, in which prevalence of HIV is high. The literacy rate is estimated to be 64% among youth in Zambia, lower than many other LMIC (Education Policy and Data Center, 2014), suggesting that incorporating audio into a computer-assisted self-interviewing system would be important in this setting.

**Fig. 1.**
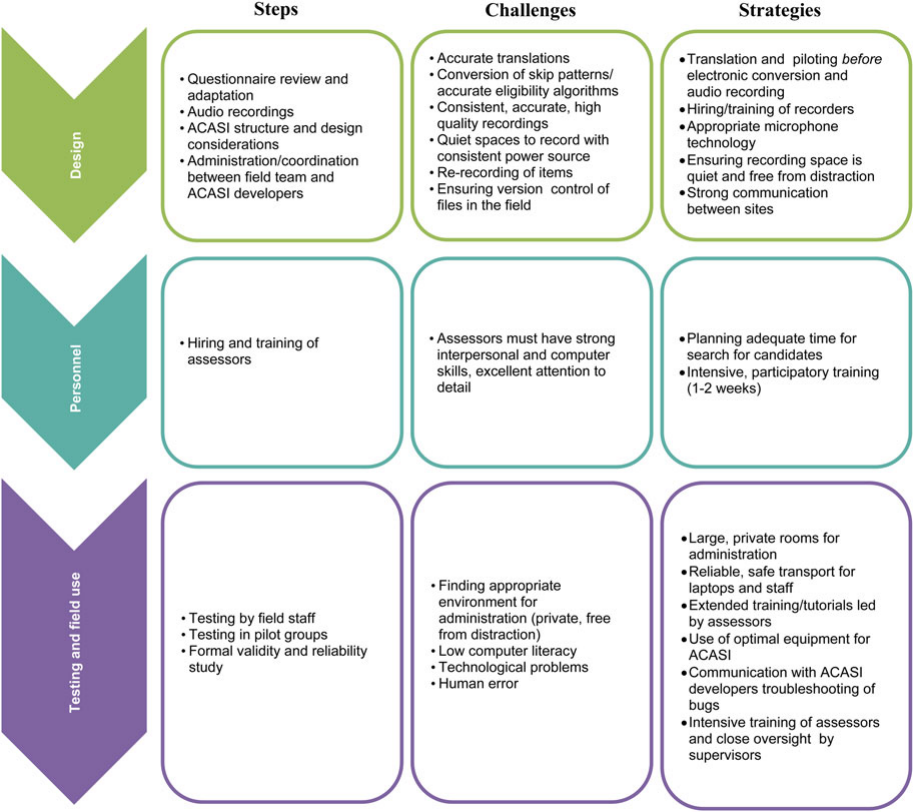
Steps, challenges, and strategies in ACASI implementation.

Our ACASI was developed for a questionnaire intended for use in a subsequent randomized trial to test the effectiveness of Trauma-Focused Cognitive Behavioral Therapy (TF-CBT) in reducing HIV risk behaviors and psychosocial problems among OVC (ClinicalTrials.gov identifier: NCT02054780). The following sections will cover the stages of ACASI development and challenges associated with each step, lessons learned, and potential strategies for addressing the challenges using Zambia as an illustrative example (see Fig. 1 for a summary).

## ACASI Development

### Structure and design considerations

A first step in development is to consider the structure of ACASI based on the instruments included in the questionnaire. We used a modular approach, which allowed each specific study instrument included in our larger questionnaire to be constructed as a singular module within the larger ACASI. Additional design specifications included: (1) a data structure outputting comma delineated text files (other options for investigators include tab or pipe-delineation); (2) an automated tutorial explaining to study participants how to answer questions and move forward; (3) a built-in algorithm determining study eligibility based on participant responses to close-ended items; (4) automated alerting of study assessors to a positive response on high risk questions (e.g. suicidal ideation); and (5) automated skip patterns and navigation based on participant responses. Box 1 summarizes equipment that we believe allows for optimal ACASI performance to incorporate these specific design specifications in a LMIC setting.

Box 1. Equipment for optimal ACASI performance in LMIC

- Laptop/tablet with password protection (ACASI software is additionally password protected)
- Laptops are portable, lightweight, and durable for trips to rural areas and have long battery life
- Matching of screen size with ACASI design. In our case this was 1024 × 600 pixels, which enabled ACASI to fill the entire screen area – important so that the participants cannot access any other computer systems or become distracted
- Software: Authorware and Audacity for voice recordings
- Headphones for clear audio and privacy
- Mouse over a touchpad: we found that participants with limited computer experience were much more comfortable using a mouse
- Touch screen: we did not have this capacity in Zambia, but future studies should investigate the acceptability of touch screen responses
- Keyboard entry capability: our ACASI allowed for participants responding by clicking on a response option using the mouse or using the keyboard to enter the corresponding number or letter to their answer. Although most used the mouse, having the keyboard entry available as an option was helpful for some participants

Our ACASI was housed on small notebook laptops. Although we expect that many researchers will begin using tablets instead of laptop notebooks in future studies, we believe that many of the challenges we experienced, such as issues of battery life and poor internet connectivity, will persist. Technology changes rapidly, but regardless of the device chosen by investigators to host ACASI (notebook, netbook, tablet), continuous power in many LMIC settings is an issue. In choosing the device, we suggest that investigators consider how it will be used both during and after the study (i.e. for future studies). Although some tablets may have better battery life than notebooks, there are a number of factors that affect battery degradation. If notebooks are used, there is the ability to purchase spare batteries and insert them easily in the field (a rare feature for tablets). The initial lower cost of tablets may be negated if there is subsequent need for add-on keyboards or protective casings to protect the screen and the keyboard. An important consideration may be to determine how study participants in a specific setting prefer to input answers (i.e. keyboard, mouse, touchpad, touchscreen), as well as how the participants will use the device (e.g. on their lap, on a table, etc.) and select an appropriate device based on these preferences. In Zambia, we found that participants were much more comfortable using a mouse compared with a touchpad (an option that the laptop-based ACASI permitted). More laptops are coming with touchscreens, and can be configured to work as a tablet or a traditional laptop, depending on the positioning of the screen. With that in mind, a touchscreen laptop could ultimately be more cost effective compared with buying a tablet, keyboard, stand/holder and protector separately. Additional details on our ACASI structure itself and design considerations for the Zambia study, including screen shots of questionnaire items, are included in online Supplemental File 1.

### Questionnaire review and adaptation

Our measure included over 250 items, several different question types with skip patterns, and was translated into the two most commonly spoken local languages in Zambia, Bemba (spoken by 33.5%) and Nyanja (spoken by 14.8%) (Central Statistical Office, 2012). Our measures were translated and back translated by a professional document translation service in Lusaka. Different translators were used for the translation and back translation; translators met with a third party to resolve discrepancies as recommended by the World Health Organization (2016). It is critical to not only have the English paper version of the measure translated and back-translated, but also reviewed and pilot tested with the target population before starting the audio recording process of ACASI. For example, in Zambia, our pilot of the paper measure revealed that approximately 15 items (6% of all items) in the Nyanja language version were viewed by participants as ‘too heavy’ or ‘too formal.’ Although technically accurate, the translations used a formal version of Nyanja not often spoken among young people in Lusaka, so these were altered on paper before audio recording. If these steps are not done well, challenges will occur with needing to modify both the electronic version of the text (in each language) and the audio files themselves (see below). All skip patterns must also be reviewed and accurate on paper in all languages before building the logic into an electronic version or participants may be asked incorrect or non-applicable questions in ACASI.

### Audio recordings

Once the paper instruments are finalized, audio recordings are needed for each individual item. Staff hired at the beginning of our project as study interviewers and M&E staff were also asked to record audio files. We learned important lessons within this audio recording stage. First, training recorders to speak slowly, clearly, and recite verbatim the text of each question and item response as written is a critical factor in the success of the audio recordings. Verifying the accuracy and pronunciation with which the recorder speaks the language can be done via a similar process as testing text questions with pilot groups; the recorder can practice reading questions aloud to pilot group participants. We did not conduct this type of pilot testing with our recorders and found during later piloting of ACASI that approximately 40 questions and response options (15% of all items) were recorded with pronunciations or accents that were unfamiliar to most Nyanja speakers. Therefore, it is important not only that a recorder is fluent in a language, but also that they enunciate the questions in a way that is understandable to the study population. Mispronunciation or unfamiliar accents could result in study participants incorrectly responding to items or responding ‘I don't know’ to items because the audio is not clear.

For future ACASI studies, we prepared a script for recorders that allowed them to read each item word-for-word and to make sure each question was recorded consistently. For example, a paper-based questionnaire may refer to ‘…the questions below’ but when adapting this to ACASI the recorder might say ‘…the following questions’ or ‘…the questions on the following screens.’ The script ensures that recorders are doing this in a consistent manner.

Second, we learned that recordings had to be free of any background noise. We had challenges finding space within our study office in Lusaka that had a consistent power source (for laptop charging during recordings) and also completely free of background noise. The office is in an urban location and home to several ongoing projects and over 30 employees. The temperature in Lusaka is typically hot and there is no air conditioning in the building. The windows are thus always open and the background noises we encountered were often related to voices of staff members in the building and traffic noise from pedestrians and cars in the street. Approximately 50 questions and response options (approximately 19% of all items) had to be re-recorded because of background noise or otherwise insufficient clarity of the recording. These re-recordings resulted in delays and additional cost of person-time devoted to the re-recordings and re-programming of audio files into ACASI. In order to reduce background noise, we had staff record at the beginning and end of the day and on weekends, when the building and surrounding area was quieter. Having a technician readily available to assess the quality of the recording in real time is also helpful for expediting re-recordings.

Third, in order to download the Audacity program and transfer audio files to the programming team based in the USA, consistent internet connection was needed. We often did not have consistent internet and speeds were slow (average internet speed in Zambia is <1 mbps) (Net Index, 2011), resulting in delayed and sometimes incomplete transfer of files. This was a time consuming process given that we had to transfer approximately 840 audio files of approximately 2 mb each to the ACASI programming team. This could be aided in future studies with pre-planning to download and transfer from locations with fast internet (e.g. many hotels in Lusaka) or at particular times (early morning or late evening) when connectivity is generally better. Re-recording of items due to translation issues, background noise, pronunciation, mismatched audio with text, or inaccurate recordings can all be challenges in setting up an ACASI system in LMIC.

### Administration and coordination with field team during ACASI development

To ensure coordination between USA-based ACASI developers and the field team in the Lusaka office, ACASI developers set up a Dropbox folder (dropbox.com) to share files over the internet. Version control of files is critical to ensure that the study team is using the same and most updated version of ACASI. Each modular file was embedded with a version date for tracking purposes. This version date was also included in the data output so that the data could be easily traced back to the specific sets of ACASI files that generated that set of data. However, close coordination between sites remained important given difficulties with internet connections in Zambia. For example, Dropbox folders would sometimes take hours or even days to update in Zambia due to internet shortages and/or the large size of ACASI system files. The ACASI programming team converted .wav files that were recorded in Zambia to .swa files in order to reduce the size of ACASI and improve transfer times. Overall, the use of a cloud-based server such as Dropbox was a success and enabled the team to exchange files such as text, audio, and ACASI software files across the globe.

## Testing and piloting ACASI

As a first step, we found that it was important for ACASI to be tested by members of our research team. This included nine testers: one of the principal investigators, a co-investigator, five study assessors (study interviewers who facilitated the ACASI assessments), one M&E officer, and the Study Director. Every possible skip pattern, audio recording, and text needed to be checked in all languages to ensure proper functionality.

In a second step, we convened pilot groups of community members. This consisted of a convenience sample of 24 participants from the same source population as our planned randomized trial. Community members in three Lusaka neighborhoods were recruited by Home Based Care Workers (HBCWs) who provide services to HIV-affected families in their communities. We asked HBCWs to refer adolescents who were between 13 and 17 years old who they believed met World Health Organization criteria for orphans or vulnerable children (part of our trial inclusion criteria). Caregivers of these adolescents were also recruited.

ACASI pilot groups are important because they provide instructive information beyond the piloting of the paper-based questionnaire. Not only do these groups provide additional feedback on the appropriateness of the survey itself (e.g. with regard to translation accuracy and comprehension) but also on aspects of laptop-based surveys and ACASI specifically. These groups were convened to obtain feedback on the translation accuracy of the questionnaire and functionality of the ACASI system. We did not retain any additional data on these community members.

Each group was facilitated by a study interviewer (assessor). Interviews took place in private rooms at local parishes in the community of the participants. All participating adolescents were provided a laptop, mouse, and headphones. Following instruction from the assessor, each adolescent individually began the interview on their laptop using ACASI. After the end of each interview section, the group would reconvene and the facilitator would ask for feedback on: (1) translation accuracy; (2) accuracy of voice recording and matching with screen text; and (3) degree of difficulty with navigating the interview on the laptop. Community participants were asked for verbal feedback after each section on any concerns or mistakes that they encountered in ACASI (participants could also take notes on a provided piece of paper while completing the interview). The assessor kept comprehensive notes during the session. Following the pilot group, the notes from the session were summarized in a Microsoft Excel document.

Our ACASI pilot groups in the community revealed the need for additional refinement in Nyanja translations and pronunciations for 40 items – changes that had not been indicated by our pilot with the paper measure. Precise recording of items was critical because we also learned from our pilot groups that many participants were relying more heavily on the audio than the text when responding to questions. The pilots also provided useful information on the computer literacy of our target population. We found that the adolescents, even including many of those who had never used a computer previously, were able to quickly acquire computer skills (e.g. using the headphones, mouse, keyboard) with only a brief tutorial. Most adolescents in our pilot groups functioned completely independently during the interview itself, only needing clarification on question content, not computer assistance. We learned that the caregivers often required more training on computer use before being able to complete the interview independently and more frequent assistance from study assessors on computer skills during the interviews.

Given the unique information provided from this phase, we believe that it is crucial that piloting occur at two stages: after the development of the paper questionnaire (before it is converted to ACASI) and following the building of the system. This two stage testing provides important feedback on both the content and translation of the questions (phase 1) and the functionality and acceptability of the ACASI version of the questions (phase 2).

## Implementation for field use

### Hiring and training of assessors

The role of study assessors is different when using an ACASI interview compared with a paper-based questionnaire. However, we learned that when hiring for this position it was equally important to identify individuals with similar skill sets as traditional interviewers: strong interpersonal skills, attention to detail, fluency in English and at least one other study language, and the ability to perform tasks consistently across participants. Additionally, we found that assessors in the ACASI study had to possess very strong computer skills. The assessors were expected to: (1) navigate the ACASI interview themselves; (2) use the higher level ACASI control functions; (3) set up and close out of ACASI sessions; (4) explain instructions for each interview section to participants; (5) help participants with navigating through ACASI; and (6) address any computer or technical issues (e.g. recovering data files if laptops shutdown partway through an interview).

Study authors (J.K., J.D., S.S.v.W.) conducted an initial 4-day ACASI training for our assessors at the study office with intermittent refresher trainings following the commencement of the randomized trial thereafter. The first day of the initial training was primarily didactic instruction, including the research aims and study design, introduction to the questionnaire and instructions for each section that they would be covering with study participants, and a walk-through of ACASI and its control functions. The second day included tutorials of using the laptops and practice-based sessions in which the assessors independently completed an ACASI interview.

The final 2 days were devoted to mock assessments where the assessors practiced all phases of study interviews–recruitment, consent, and ACASI interview including: (1) laptop and ACASI set-up, (2) training participants on laptop use, (3) administering instructions for each interview section, (4) assisting with laptop or interview questions from participants, and (5) successful ACASI close-out. We presented the assessors with real-world scenarios based on situations that arose during our initial ACASI pilot groups (e.g. older participants with difficulty using the mouse, participants accidentally exiting ACASI). Each morning of the training we had a formal quiz as well as a final post-training assessment to ensure the information provided to the assessors was retained. Following the training, assessors were closely supervised during field activity in a feasibility study (see below). Based on our experience, a 4–5 day training followed by a week of close supervision in the field is sufficient for allowing assessors to begin independently administering ACASI to study participants.

### Lessons learned through a validity, reliability, and feasibility study

As a final step before using our ACASI measure in the randomized trial, following the training of our study assessors we conducted a formal validity, reliability, and feasibility study of the measures included. We recruited 210 adolescents and their caregivers from our target population in Lusaka neighborhoods who would meet criteria for the upcoming trial (exhibiting HIV risk behaviors or symptoms of psychosocial problems, ages 13–17, and meeting World Health Organization definition of an orphan or vulnerable child). This recruitment was conducted by HBCWs, similar to our previously described pilot groups. Adolescents and caregivers completed the entire ACASI interview as they would in our trial. Although the primary purpose was to validate the measures, we also solicited informal feedback on their experience using ACASI. Assessors solicited this feedback from participants privately following the end of the interview. The study was approved by the Johns Hopkins Bloomberg School of Public Health IRB and the University of Zambia ethical review board. Assessors obtained informed consent from all participants before commencing ACASI interviews. The following are lessons learned from ACASI implementation within this study.

Environment of administration is an important consideration. Although the interview is self-administered with the use of headphones for additional privacy, the interviews should be conducted in spaces where the participant feels comfortable responding to sensitive questions and the risk of confidentiality breach is as low as possible. We conducted our study in various churches and parishes throughout the Lusaka study sites. Studies were conducted at these locations because they provided large rooms where participants could simultaneously complete ACASI but have sufficient space between participants so that no one else could view their screen or hear their audio. Flat surfaces for the laptops and sufficient space to operate the mouse are also required. Additionally, care must be taken in arrangement of safe transport of equipment between field sites for interviews and study offices. Our study team was responsible for transporting approximately 20 laptop computers from the study office in Lusaka to the parish in the study community on a daily basis. It was therefore critical that our study transport vehicles were readily available and drivers were on time to pick up the team and equipment at the end of each day. We did not employ security staff for this study but it may be advisable in areas where crime and safety are very serious concerns and/or after dark.

It is important to find a balance between providing participant autonomy and privacy and having an assessor stationed nearby in case of difficulty with ACASI. Based on our original pilot groups where some participants had difficulty using laptop computers, we determined that the assessor would sit with all of the participants for the first ACASI section, which measured demographic characteristics. As these questions were relatively innocuous, concerns about sensitivity or social desirability bias were minimal. The demographics section therefore functioned as both an interview section and an extended tutorial for participants who needed extra time to become familiar with ACASI laptop functionality and navigation. Following the demographics section, the assessor explained instructions for the upcoming questions and instructed the participant to notify him/her with any questions. The assessor remained in the room and could be easily alerted by a raise of the participant's hand, but far enough away from the participant so as to not be able to hear the audio or view the laptop screen. Participants did not express any privacy concerns while completing interviews or when asked about their experience afterwards.

The response from participants after using ACASI was positive, and many reported to our assessors that the laptops were not only easy to use, but enjoyable. This was particularly the case among adolescent participants who typically did not have the opportunity to use laptop computers. Some elderly participants were initially hesitant and/or intimidated to use a computer, with one participant expressing, ‘I am from the old times.’ In these circumstances, assessors offered more extensive demonstrations with the computer, mouse, and headphones, and encouraged participants to type on the keyboard, click on the mouse, and familiarize themselves with the new technology. Although this increased the time necessary to complete the interviews, in the majority of these instances, participants who were provided this encouragement and support eventually became comfortable, and reported having enjoyed completing the interview and learning a new skill (i.e. how to use a computer for the first time). In cases of very low or no literacy or computer ability, the assessor would complete the interview alongside the participant, although this happened rarely. In these cases the participant would listen to the questions as presented by ACASI audio, and the assessor would input the participant's response and control navigation of the interview.

Specific ACASI design functions were helpful in the field and reduced burden on the assessors. ACASI was able to automatically produce eligibility status for study participants precluding the need for assessors to determine this manually. Random quality checks on the automated eligibility function found that the system determined eligibility with 100% accuracy. Retrieval of study data at the end of each day was expedited by the organization of the data text files stored on each laptop. Incomplete interviews were stored within an incomplete data folder on each laptop and were completed where the participant left off at a later date with no problems. Perhaps most importantly, ACASI successfully identified and flagged participants with high risk for possible suicidal ideation or intent based on their responses to specific questions. Following the end of the interview, ACASI alerted the assessors automatically via a password protected screen if the participant had indicated suicidal ideation during the questionnaire. The assessors were then able to immediately call our clinical supervision team who then did further evaluation and took action in accordance with the study safety protocol.

There were some technical issues that arose with the laptops and ACASI such as laptops shutting down without warning, non-working mice and headphones, and fast-draining batteries. These primarily were the result of using laptops in temperatures between 85°and 90° Fahrenheit, and in dusty settings with unreliable power sources. Although not used in our study, investigators working in rural Zimbabwe, recharged laptop batteries by connecting them to the battery of a truck, which was powered by a solar panel (Langhaug *et al.* 2011), and this may be an option in future research. Still, the potential for technological issues in the field is likely to be present in LMIC settings. It is therefore important for assessors to be nearby during participant interviews and for them to be functional in troubleshooting basic computer problems, which arose in our study on a daily basis. Communication between the field team and ACASI programmers is a key to facilitate solutions to any ACASI bugs that occur.

The most frequent issues in ACASI implementation were not associated with technological challenges, however, but with human error. Repetition with administering ACASI became tedious over time for assessors. Common mistakes included: (1) entering incorrect study ID numbers into ACASI; (2) incorrect or insufficient tracking of study laptops, which was particularly problematic for incomplete interviews that had to be completed on the same laptop at a later date; and (3) recording the wrong eligibility status displayed by ACASI onto a client intake form (i.e. mistaking an eligible client as ineligible or vice versa). It was important therefore to keep assessors motivated and to treat each and every interview with the same high level of attention to ensure quality of data. It also required close oversight of assessors by the research team in the study office at the end of each day and regular data quality checks. For example, extraction of data from field laptops to the main office-based study computer at the end of each day required meticulous attention to detail (see online Supplemental File for process of extraction). This responsibility was delegated to trained data staff in the study office and required weekly quality control checks to ensure that data transfers were accurate and complete.

## Limitations

This investigation had several limitations. First, we did not conduct a direct comparison study between ACASI and other assessment modalities, such as self-administered questionnaires or face-to-face interviews. Many other studies cited in this paper have made such comparisons; our goal was to present the process of developing an ACASI for a LMIC and the challenges associated with that process. Second, although we solicited informal feedback from participants during the reliability, validity, and feasibility study, we did not record specific numbers or counts on issues that participants reported while they completed ACASI. That information would have provided more precise information on the degree to which problems were encountered. Finally, we did not conduct a formal cost-effectiveness study for this ACASI and cannot determine to what degree (if at all) it would have been more cost effective than a paper-administered questionnaire.

## Conclusions

The use of electronic data collection through ACASI has increased substantially in LMIC (Langhaug *et al.* 2010), a trend that is likely to continue over time and perhaps become a new standard in behavioral health and clinical research. Studies have indicated that ACASI is preferable to self-administered questionnaires and face-to-face interviewing because of improved data quality, improved experience of study participants, reduction in burden on study personnel (i.e. data entry) , and potentially reduced long-term costs (Langhaug *et al.* 2010; Brown *et al.* 2013). This paper reported on the steps for development, testing, and implementation of ACASI within a LMIC setting to help researchers and programs plan for and utilize this system more efficiently.

Our experience in Zambia suggested that: (1) a lengthy and complex questionnaire with many skip patterns, eligibility algorithms, and design specifications can be accurately translated from a paper version in multiple languages to an electronic ACASI; and (2) the ACASI version of the questionnaire can be implemented by trained staff and is likely to be acceptable to study participants if training and support are supplied by assessors. However, we found that there were challenges specific to this LMIC setting relative to high income countries. These included: translating and recording the questionnaire in multiple local languages, implementing ACASI among populations with limited computer literacy, and troubleshooting computer problems in resource limited settings. Suggestions for future users include: (1) piloting after the development of the paper questionnaire and following the building of ACASI; (2) adequate training for ACASI assessors and voice recorders; (3) regular oversight of assessors to minimize human error and attention to safety given use and transport of electronics, and (4) consideration of environment of implementation (e.g. private locations).

Even with ACASI challenges, we believe that ACASI systems are a strong alternative in LMIC research settings. It remains important to consider implementation factors in any particular LMIC such as acceptability, feasibility, cost, and scale-up potential. Our challenges became lessons learned, which hopefully will reduce the cost and time required to use ACASI in LMIC for future researchers and programs. Additional implementation research, such as mixed methods interviews with various levels of stakeholders (e.g. study participants, ACASI programmers, field research team) and formal cost effectiveness analyses focused on dissemination and implementation will enrich the literature on ACASI in low resource contexts and improve our ability to design and use better methodological tools for analyzing important and sensitive health outcomes. Finally, additional papers on the actual implementation process of using systems like ACASI are needed so researchers and programs can make more informed choices.

## Supplementary material

For supplementary material accompanying this paper visit http://dx.doi.org/10.1017/gmh.2016.19. click here to view supplementary material

## References

[ref1] Adebajo S, Obianwu O, Eluwa G, Vu L, Oginni A, Tun W, Sheehy M, Ahonsi B, Bashorun A, Idogho O, Karlyn A (2014). Comparison of audio computer assisted self-interview and face-to-face interview methods in eliciting HIV-related risks among Men Who Have Sex with Men and Men Who Inject Drugs in Nigeria. PLoS ONE 9, e81981. 24416134 10.1371/journal.pone.0081981 PMC3885382

[ref2] Aquilino WS (1997). Privacy effects on self-reported drug use: interactions with survey mode and respondent characteristics. NIDA Research Monographs 167, 383–415. 9243571

[ref3] Beauclair R, Meng F, Deprez N, Temmerman M, Welte A, Hens N, Delva W (2013). Evaluating audio computer assisted self-interviews in urban south African communities: evidence for good suitability and reduced social desirability bias of a cross-sectional survey on sexual behaviour. BMC Medical Research Methodology 13, 11. 23368888 10.1186/1471-2288-13-11 PMC3568408

[ref4] Boris NW, Brown LA, Thurman TR, Rice JC, Snider LM, Ntaganira J, Nyirazinyoye LN (2008). Depressive symptoms in youth heads of household in Rwanda: correlates and implications for intervention. Archives of Pediatric and Adolescent Medicine 162, 836–843. 10.1001/archpedi.162.9.836 18762600

[ref5] Brown JL, Swartzendruber A, DiClemente RJ (2013). The application of audio computer-assisted self-interviews (ACASI) to collect self-reported health data: an overview. Caries Research 47, 40–45. 24107606 10.1159/000351827 PMC4511474

[ref6] Brown JL, Vanable PA, Eriksen MD (2008). Computer-assisted self-interviews: a cost effectiveness analysis. Behavior Research Methods 40, 1–7. 18411521 10.3758/brm.40.1.1 PMC2572260

[ref7] Central Statistical Office (2012). 2010 Census of Population and Housing: National Analytic Report. (https://web.archive.org/web/20131217101229/http://www.zamstats.gov.zm/report/Census/2010/2010%20Census%20of%20Population%20National%20Analytical%20Report%20-%202010%20Census.pdf). Accessed 17 May 2016.

[ref8] Cluver L, Orkin M (2009). Cumulative risk and AIDS-orphanhood: interactions of stigma, bullying and poverty on child mental health in South Africa. Social Science and Medicine 69, 1186–1193. 19713022 10.1016/j.socscimed.2009.07.033

[ref9] Des Jarlais DC, Paone D, Milliken J, Turner CF, Miller H, Gribble J, Shi Q, Hagan H, Friedman SR (1999). Audio-computer interviewing to measure risk behaviour for HIV among injecting drug users: a quasi-randomised trial. Lancet 353, 1657–1661. 10335785 10.1016/s0140-6736(98)07026-3

[ref10] Education Policy and Data Center (2014). Zambia. (http://www.epdc.org/sites/default/files/documents/EPDC%20NEP_Zambia.pdf). Accessed 17 May 2016.

[ref11] Ghanem KG, Hutton HE, Zenilman JM, Zimba R, Erbelding EJ (2005). Audio computer assisted self interview and face to face interview modes in assessing response bias among STD clinic patients. Sexually Transmitted Infections 81, 421–425. 16199744 10.1136/sti.2004.013193 PMC1745029

[ref12] Langhaug LF, Cheung YB, Pascoe S, Hayes R, Cowan FM (2009). Difference in prevalence of common mental disorder as measured using four questionnaire delivery methods among young people in rural Zimbabwe. Journal of Affective Disorders 118, 220–223. 19303145 10.1016/j.jad.2009.02.003 PMC2745501

[ref13] Langhaug LF, Cheung YB, Pascoe SJS, Chirawu P, Woelk G, Hayes RJ, Cowan FM (2011). How you ask really matters: randomised comparison of four sexual behaviour questionnaire delivery modes in Zimbabwean youth. Sexually Transmitted Infections 87, 165–173. 20943824 10.1136/sti.2009.037374

[ref14] Langhaug LF, Sherr L, Cowan FM (2010). How to improve the validity of sexual behaviour reporting: systematic review of questionnaire delivery modes in developing countries. Tropical Medicine & International Health 15, 362–381. 20409291 10.1111/j.1365-3156.2009.02464.x PMC3321435

[ref15] Metzger DS, Koblin B, Turner C, Navaline H, Valenti F, Holte S, Gross M, Sheon A, Miller H, Cooley P, Seage GR (2000). Randomized controlled trial of audio computer-assisted self-interviewing: utility and acceptability in longitudinal studies. American Journal of Epidemiology 152, 99–106. 10909945 10.1093/aje/152.2.99

[ref16] Net Index (2011). Household Internet speeds for 25 African countries, cities. (http://www.oafrica.com/broadband/household-internet-speeds/). Accessed 17 May 2016.

[ref17] Pufall EL, Robertson L, Mushati P, Sherr L, Nyamukapa C, Gregson S (2014). Protective Effect of School Enrolment Against Substance Abuse in Orphans and Vulnerable Children. Imperial College London: London (http://webcache.googleusercontent.com/search?q=cache:48tadJIlfScJ:www.riatt-esa.org/sites/default/files/PolicyBrief_substanceuseandeducation%2520final.docx+&cd=1&hl=en&ct=clnk). Accessed 15 January 2016.

[ref18] Tufts University School of Medicine (2014). Audio Computer Assisted Self-Interviewing Software. Tufts University: Boston, MA (http://acasi.tufts.edu/). Accessed 15 January 2016.

[ref19] Turner CF, Ku L, Rogers SM, Lindberg LD, Pleck JH, Sonenstein FL (1998). Adolescent sexual behavior, drug use, and violence: increased reporting with computer survey technology. Science 280, 867–873. 9572724 10.1126/science.280.5365.867

[ref20] UNICEF (2013). *Towards an AIDS-Free Generation-Children and AIDS: Sixth Stocktaking Report, 2013* (http://www.avert.org/children-orphaned-hiv-and-aids.htm#sthash.UT2jNnZs.dpuf). Accessed 15 January 2016.

[ref21] van de Wijgert J, Padian N, Shiboski S, Turner C (2000). Is audio computer-assisted self-interviewing a feasible method of surveying in Zimbabwe? International Journal of Epidemiology 29, 885–890. 11034973 10.1093/ije/29.5.885

[ref22] World Bank (2015). Zambia. (http://data.worldbank.org/country/zambia#cp_wdi). Accessed 17 May 2016.

[ref23] World Health Organization (2016). Process of translation and adaptation of instruments. (http://www.who.int/substance_abuse/research_tools/translation/en/). Accessed 17 May 2016.

