# Supplementary material for: Process and implementation of Audio Computer Assisted Self-Interviewing (ACASI) assessments in low resource settings: a case example from Zambia
Source: Glob Ment Health (Camb). 2016 Aug 12;3:e24. doi: 10.1017/gmh.2016.19 (PMC5454788; doi:10.1017/gmh.2016.19)

**Supplemental File 1: Specific design considerations for Zambia ACASI**

***Questionnaire preparation and transfer to electronic version***

The study instrument to be used in the randomized controlled trial included several primary (HIV risk behaviors, substance and alcohol use) and secondary (trauma symptoms, internalizing and externalizing symptoms, functioning) outcomes, as well as a number of putative mediators and moderators. The instrument for adolescent participants comprised 12 sections and over 350 individual items. Additionally, we developed a questionnaire for the adolescent’s parents or caregivers, which included 8 sections and over 200 items. The measures were developed in English and were also translated into two local languages (Nyanja and Bemba). In order to ensure accuracy of translation, the measures were also back-translated and then piloted in the community.

The process of converting a paper-based questionnaire to an electronic questionnaire required multiple steps. The first step was an intensive review of the instrument itself. The text in the paper version was thoroughly examined for the overall number of questions (which will have implications for cost), the types of questions (e.g., open-ended, binary, scale), the complexity of skip logic required, and finally the data output that was ultimately required. It was also critical to review the questionnaires in each required language to ensure that each version was equivalent. All of these factors were integral in determining how long it took to build the ACASI, how the text for questions and response options were displayed on the screen and the corresponding audio files recorded, how participants navigated through the questionnaire, and how data were stored.

It was therefore important to review the overall number and types of questions to reduce participant burden before translating the questionnaire to an electronic version—general survey design best practices still apply with ACASI as they do with paper versions. Participants may lose focus and attention as the number of items increases. Further, items with too many answer choices can be confusing or intimidating to participants so it may be necessary to restructure some questions for simplicity and clarity. Some questions may require pictures so it is important to plan ahead to accommodate all text and images on the screen while maintaining readability. From a health literacy perspective, it is important that the questions be as clear, simple, and concise as possible. All text should be written at a reading level appropriate for the intended audience.

The ACASI requires separate audio files that are recorded for each question in all languages and then response options that can be repeated across questions (e.g., “yes/no” or Likert scales). Recording audio files for the ACASI can be a straight forward process using a free open source software program, *Audacity* (Audacity Team, 2015). Local speakers of each language should be used to record the audio, which ensures the accuracy of pronunciation, intonation and emphasis.

Given the length and complexity of the questionnaire, we aimed to design an ACASI system that was as user-friendly as possible. We first assessed the adaptability of the text to the technology. For example, on paper a participant (or assessor) might “circle” an answer, yet on an ACASI system a participant would “click” or “select” an answer on his/her own. References such as “on next page” or “at the bottom of the page” had to be changed to “on the next screen” or “at the bottom of the screen.”

Our ACASI was developed in the three languages most commonly spoken in Lusaka: English, Bemba and Nyanja. Question text in English tended to be shorter than the text translations in Bemba and Nyanja, so it was important to plan for the maximum amount of text that would fit on the screen (while also allowing for additional space for picture representations if needed) and maintain readability before building actual questions. With that in mind, it was important to review and compare sample text in all languages for consistency and accuracy. Figure 1 displays a screenshot with the same question in English, Bemba, and Nyanja.

**Figure 1. Sample ACASI question in English, Bemba, and Nyanja languages**


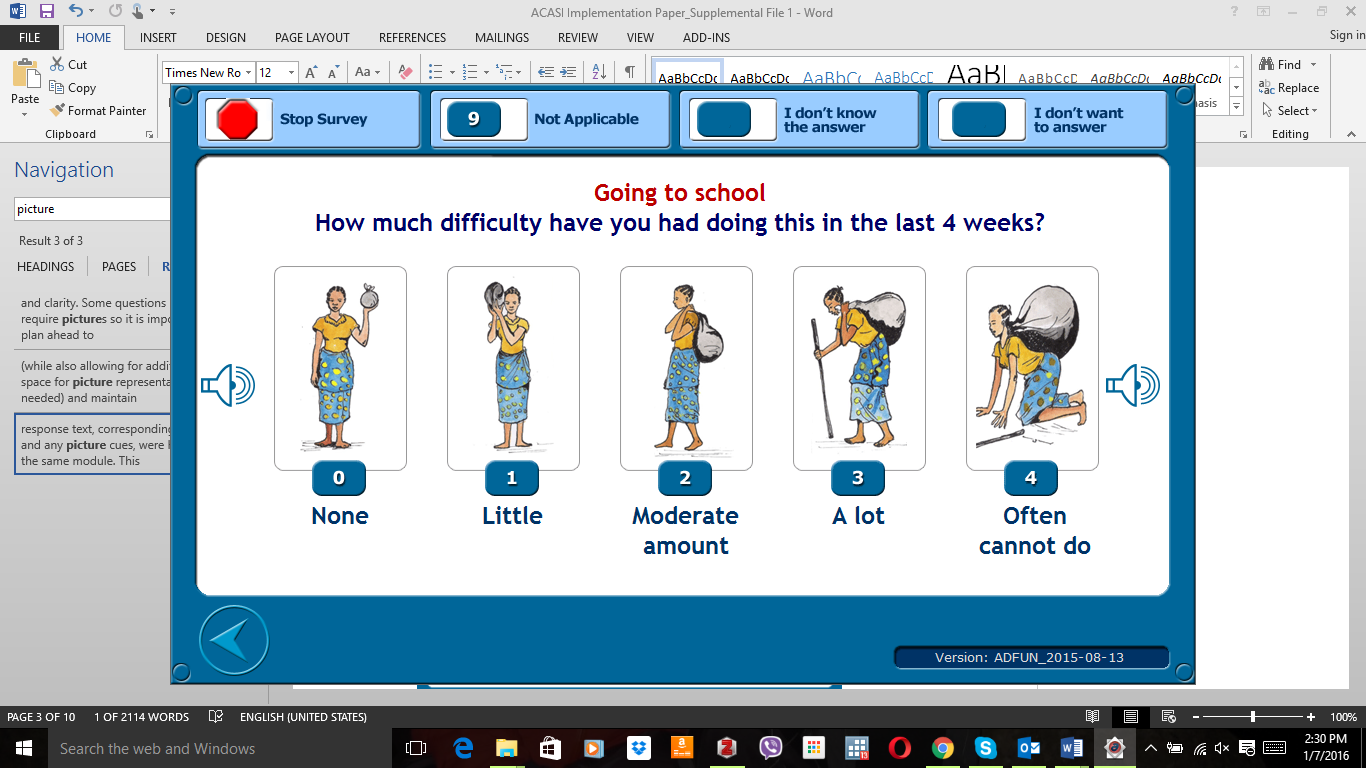


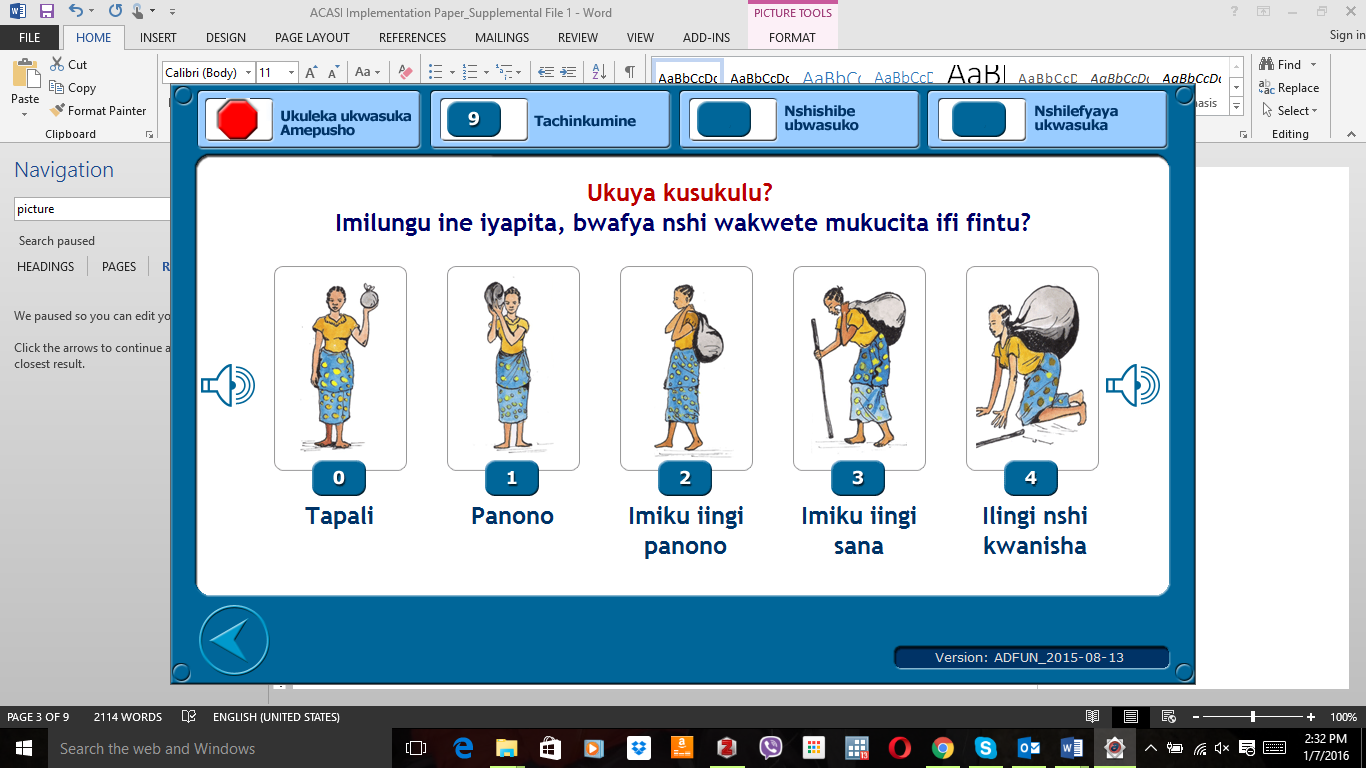


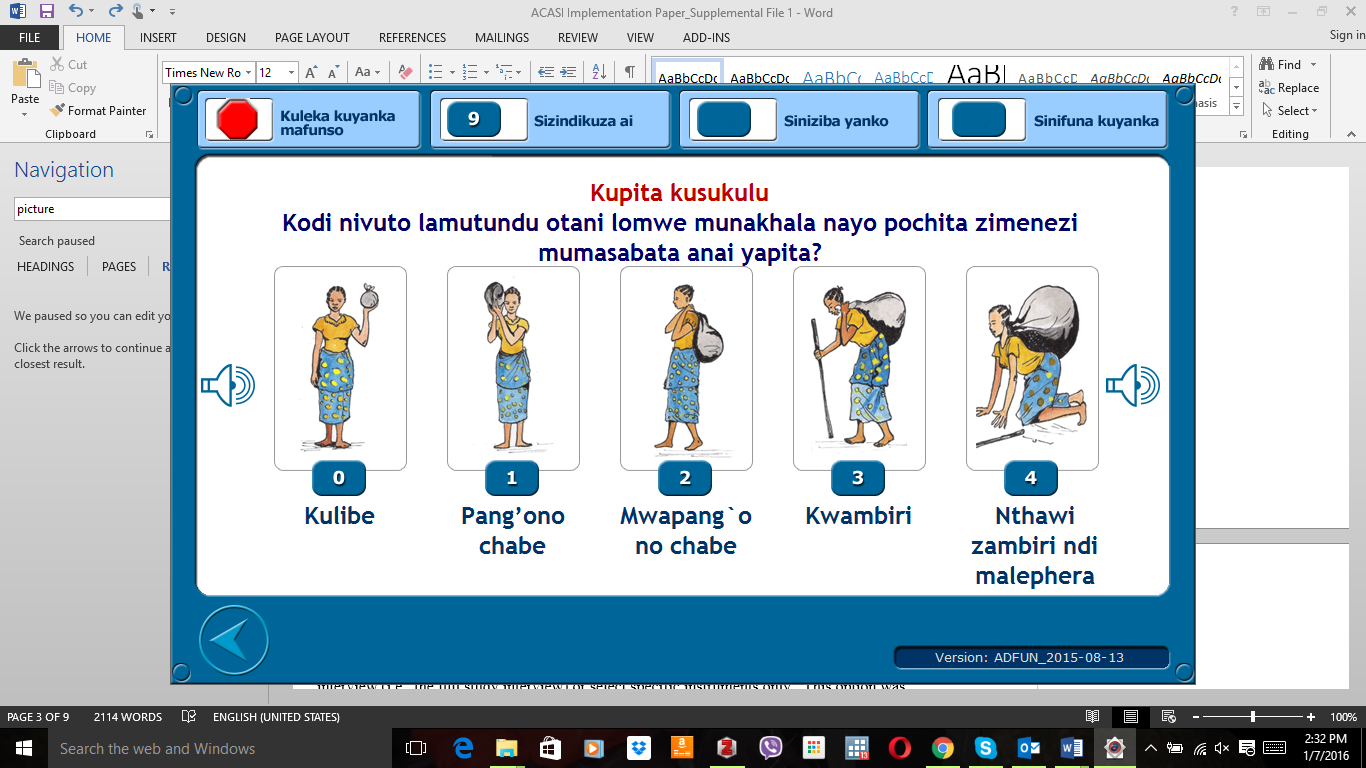


We used a modular approach in the structural development of our ACASI system. This allowed each specific study instrument included in the larger questionnaire to be constructed as a singular module within the larger ACASI. This turned out to be advantageous for several reasons. All of the information for a specific instrument (e.g., the HIV risk behavior questionnaire), including question and response text, corresponding audio, and any picture cues were housed in the same module. This allowed the ability to complete all sections of the interview (i.e., the full study interview) or select specific instruments only. This option was added to an introductory screen so that study assessors could specify when setting up the ACASI for participants if they wanted the complete interview or only certain sub-sections, displayed in Figure 2.

**Figure 2. Screen allowing assessors to choose which set of questionnaire modules to administer to study participants**


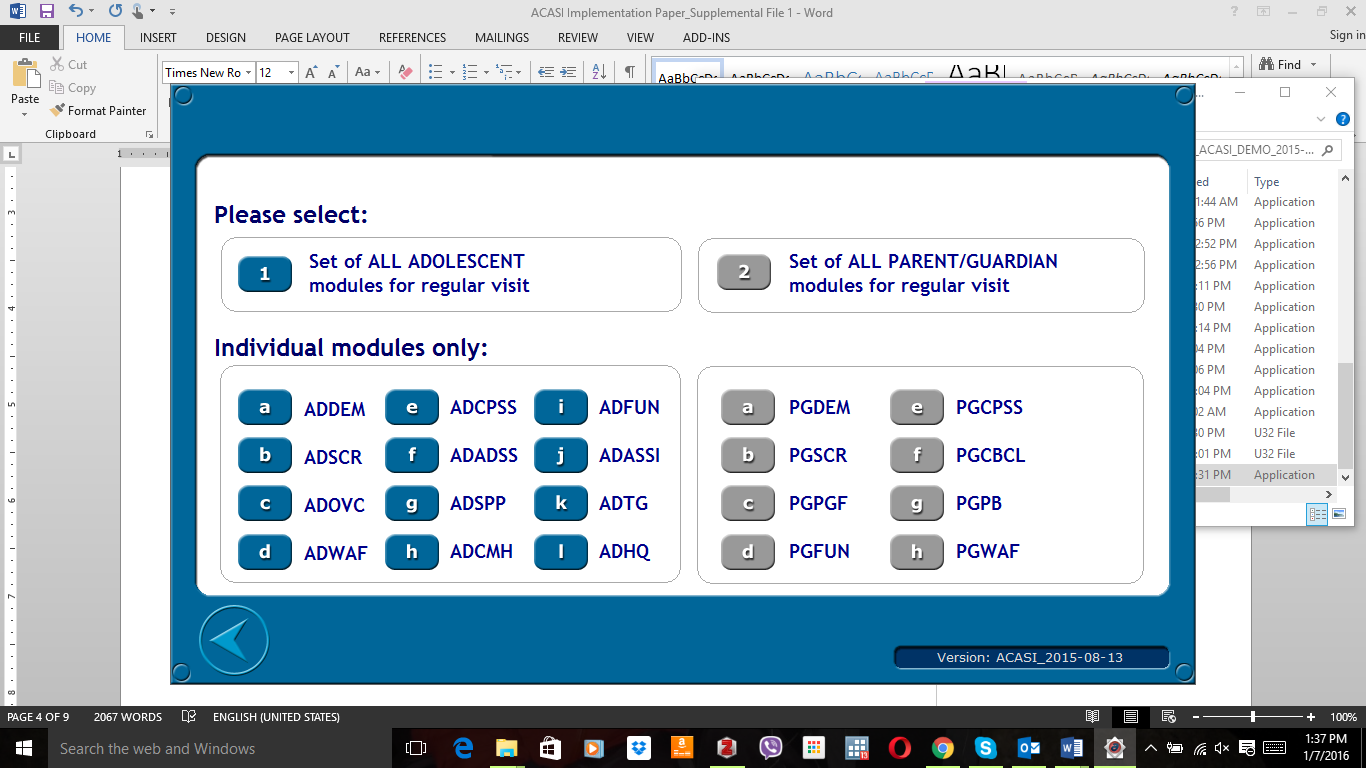


Due to the length of the questionnaire, approximately 2-3 hours, it was anticipated that some participants would not be able to complete the entire survey in one study visit. The system was developed based on discrete modules or sections; therefore the participant could stop at the end of any section and resume the questionnaire where they left off at a later date. As part of the design, section introductions and conclusions were included to help the participant navigate through the ACASI and to assist the participant in mentally transitioning from one section to another. The time in between each section also allowed for natural breaks in which we could offer the participant a snack or rest before continuing. These breaks also allowed time for the assessor to explain special instructions for upcoming sections. Password screens were inserted at the beginning of each new section which prompted participants to call over a nearby assessor; this precluded them from moving forward without explicit instruction about the upcoming instrument section. An example of the password screen is displayed in Figure 3.

**Figure 3. ACASI password screen**


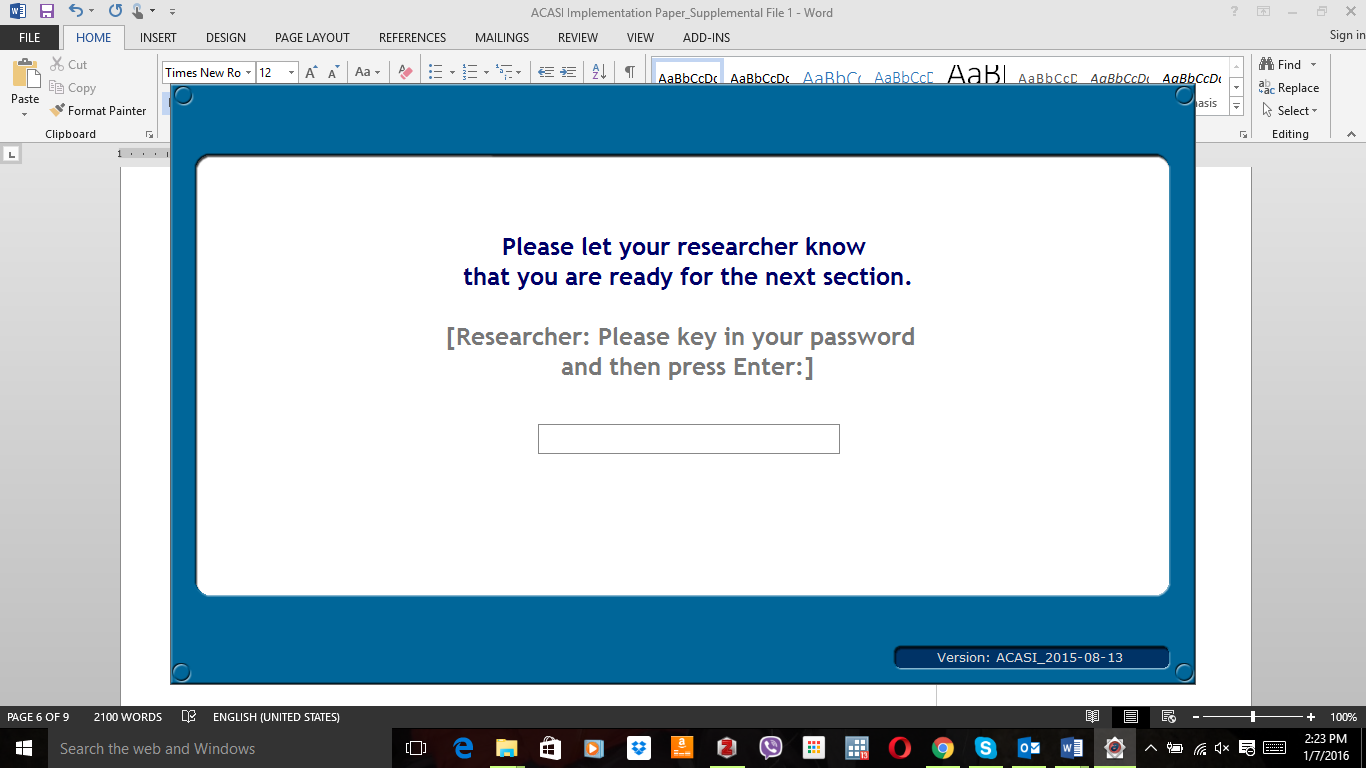


The modular structure also facilitated the ability of the field team to test the ACASI system. This approach supported incremental deliveries of the system that allowed the study team to review and test specific sections of the questionnaire (e.g., only the HIV risk behavior measure) as they became available rather than wait for the entire system to be built. Incremental versions of the ACASI were built and delivered to the study field team, section by section, language by language. Each module contained three separate versions of each question, one for each respective language. We utilized the time spent translating the paper questionnaire into Bemba and Nyanja to build an initial beta version of the full English language ACASI for review and approval. Through this design and planning, the developers were able to go back and insert Bemba and Nyanja text and audio as they became available. This enabled the team to maximize the use of available time to deliver overall functionality while meeting pressing deadlines.

***Data structure***

Data organization was critical to the overall ACASI development and storage process. Variable names were created to match sections and question numbers. For example, the variable ADDEM_01 represented the adolescent (‘AD’) demographics (‘DEM’) question 1 (‘_01’). Data were automatically stored in file folders within the larger ACASI system folder on each study laptop. Data were organized by completion status (complete or incomplete) and participant type (adolescent or caregiver). Storage of incomplete files in these folders allowed participants to continue interviews where they left off at subsequent visits.

Data were saved in comma-delimited text files and were automatically stored with the ID number as the file name. This facilitated transfer from the study field laptops to the central study database (via flash drive) at the end of every study day. The text files were stored for back up on the main study computer and were also imported into Microsoft Excel and later statistical packages for management and analysis. The versatility of the text file—it can easily be imported into most Office and statistical packages—made it an excellent choice for data structure within ACASI.

***Design considerations***

At the start of the development process, sample screens were developed for review, modification and approval. The flow of the initial screens in the ACASI included a password/login screen, custom screens for the entry and verification of a participant ID format specific for this project, as well as a screen for the interviewer to select the appropriate language. These screens were followed by a brief tutorial/orientation screen explaining to the participant how to answer questions and move forward. The tutorial provided an automated animation with accompanying audio of how each button worked and overall navigation functionality. We believed that this brief tutorial was critical given that many of our study participants would be using a laptop computer for the first time in this study (displayed in Figure 4).

**Figure 4. Introductory ACASI demo screen**


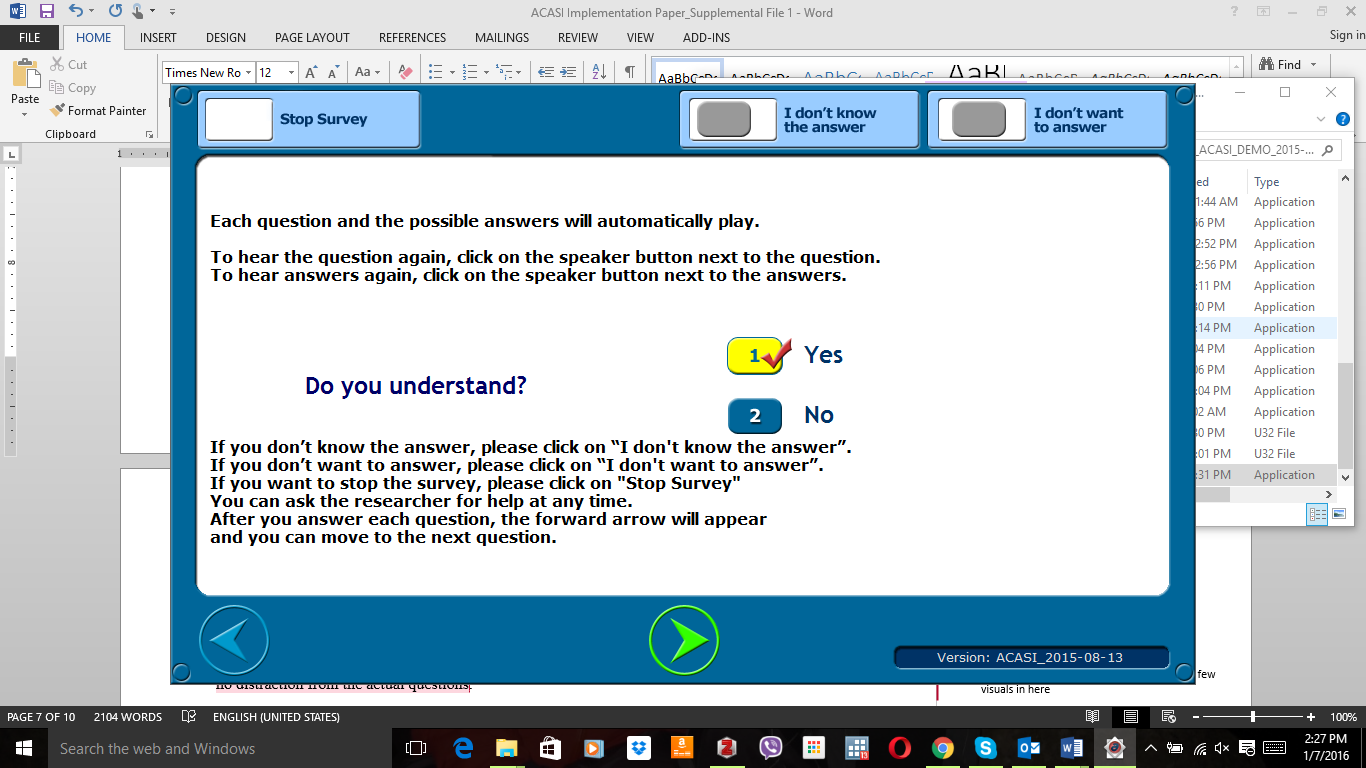


The overall design was consistent across all modules. Dark text was presented on a white background for maximum contrast. In general, large text (Trebuchet font, 18pt, bold) was used for readability. The interface design was intentionally simple and intuitive so that there would be no distraction from the actual questions.

There were a number of specific design choices made based on our particular study in Zambia that may also be relevant to behavioral health research in other LMIC settings. First, special feedback screens were developed for the study assessors to easily review the screener section that has the participants’ eligibility information. The first two sections of our interview were comprised of demographics and screener sections. Based on an algorithm of responses to these sections, ACASI was programmed to calculate a participant’s eligibility following the screener. After responding to the last question of the screener, a message would appear for the participant to call over the study assessor. The assessor entered a password and a screen indicating the participant’s eligibility was displayed. This allowed us to easily and instantly determine study eligibility of the participant and whether they should continue with other sections of the interview or not (Figure 5).

Second, we anticipated working with a population that had experienced a significant amount of trauma and may have severe mental health problems. It was therefore important for ACASI to ‘flag’ participants who may be at high risk for self-harm or other symptoms or behaviors that warranted immediate referral for service. Following the final question of the interview, the assessor would enter a password into ACASI and a summary screen would appear that included a blank circle if there was no ‘flag’ or a red circle in the case of a high risk participant. Research staff were trained on how to handle high-risk cases following the assessment. In all cases a counselor or clinical supervisor was immediately called to talk with the participant. This system allowed the participant to answer the initial sensitive questions privately (compared to an in-person interview) but also allowed for real-time follow-up in high risk cases. The eligibility and high risk screen is displayed in Figure 5.

**Figure 5. ACASI screen displaying eligibility status to the assessor**


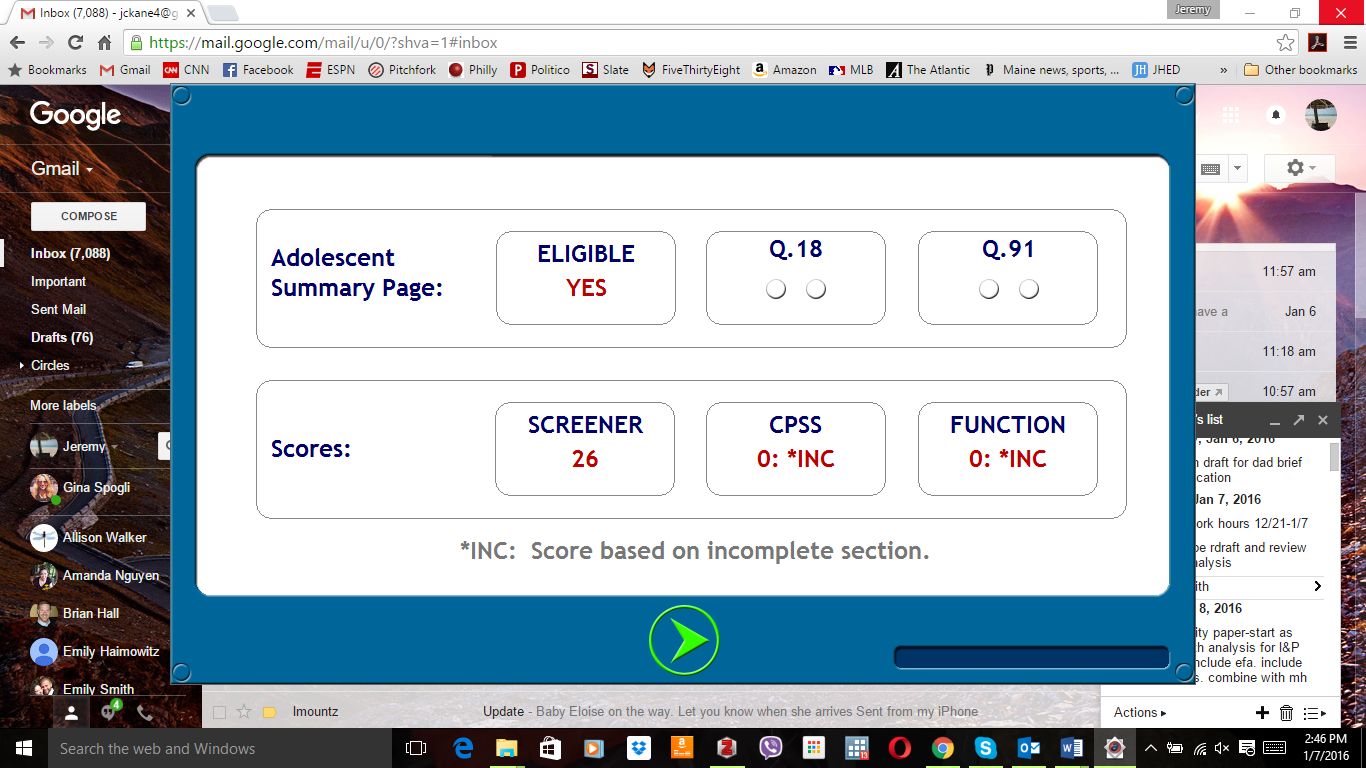


Our third specific design choice was in deciding how much control to give the study participants, including the ability to navigate, stop, refuse to answer questions, and to replay audio files. We wanted our study participants to be able to complete the interview with as much autonomy and privacy as possible, but we still wanted still have the ability to explain question types to them (hence the aforementioned password screens). We included navigation buttons on each screen which allowed the participant to move forward and backward as many times as necessary; anticipating that participants who are new to laptop use might make a mistake and want to modify their answer. We allowed participants to select an answer and move to the next question after the audio to the question was read aloud but before all of the response options were read aloud. Although many participants relied on the audio, we were concerned that having to repeatedly listen to all response options would be frustrating for highly literate participants. We therefore gave them the option of selecting a response and moving on without having to listen to the full list of response options.

Options for the participant to indicate “I don’t know the answer” and “I don’t want to answer” were also present on every question screen in addition to the question-specific response options. “Not applicable” options were also included when relevant to the question. Buttons with speaker icons were strategically placed on the screen to allow the participant to replay either the question audio file or the set of answer choices for that question. Participant controls also included a “Stop Survey” button on every question screen if they wished to stop. If this button was pressed accidentally, ACASI was able to pick up again where the participant left off.

Finally, a special set of controls reserved for use by the study team were created and accessible only through a special password-protected “Control Panel”. Options available through the control panel would allow the interviewer to be able to quit, turn audio on and off, view and hide question numbers, view and hide variable names and values, and change languages (Figure 6).

**Figure 6. ACASI screen displaying password-protected control options**


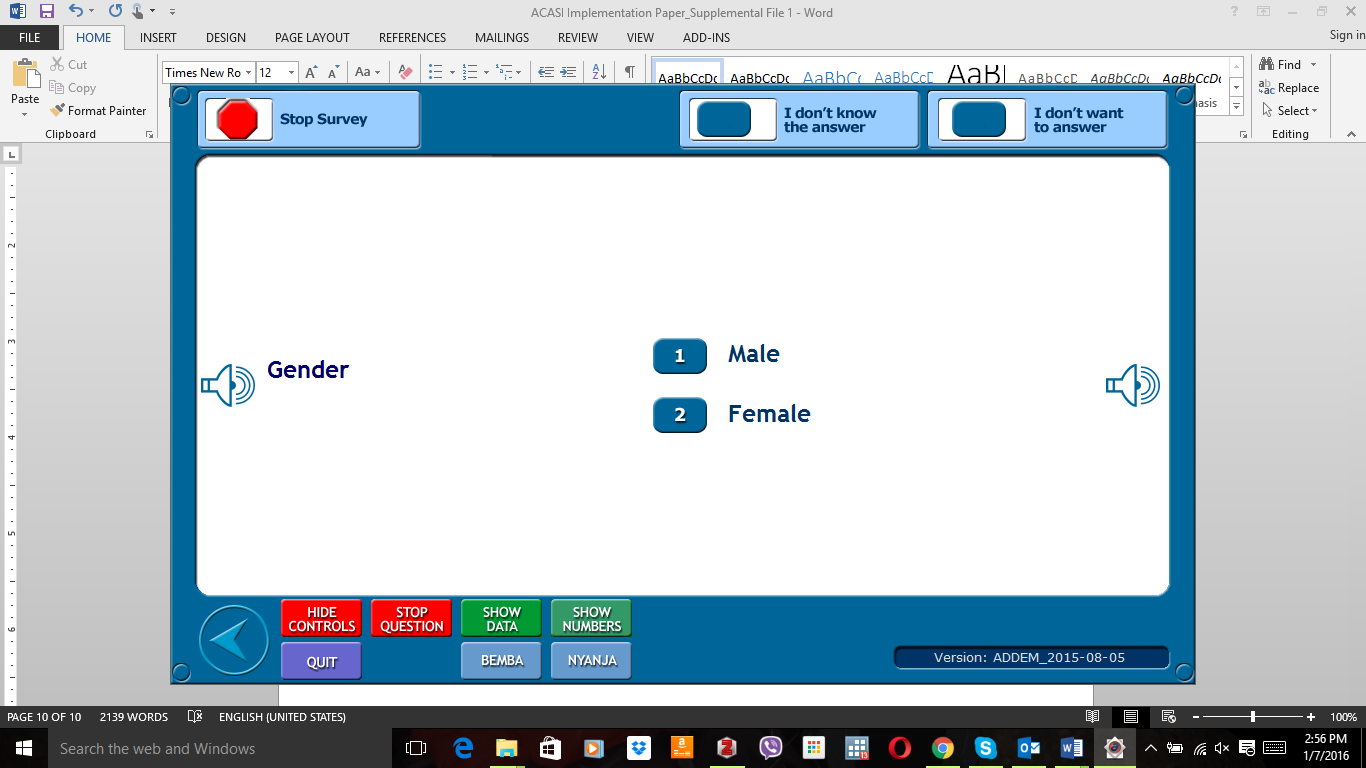

Supplement: Supplementary file 1 [file S2054425116000194sup001.docx]
